# 多肽识别导向的外泌体分离富集方法研究进展

**DOI:** 10.3724/SP.J.1123.2024.10015

**Published:** 2025-05-08

**Authors:** Kun XU, Yanyan HUANG, Rui ZHAO

**Affiliations:** 1.北京分子科学国家研究中心, 中国科学院活体分析化学重点实验室, 中国科学院分子科学科教融合卓越创新中心, 中国科学院化学研究所, 北京 100190; 1. Beijing National Laboratory for Molecular Sciences, CAS Key Laboratory of Analytical Chemistry for Living Biosystems, CAS Research/Education Center for Excellence in Molecular Sciences, Institute of Chemistry, Chinese Academy of Sciences, Beijing 100190, China; 2.中国科学院大学, 北京 100049; 2. University of Chinese Academy of Sciences, Beijing 100049, China

**Keywords:** 外泌体, 分子识别, 多肽, 分离富集, exosome, molecular recognition, peptide, isolation and enrichment

## Abstract

外泌体是由细胞分泌的双层膜囊泡,携带有丰富的生物分子,是细胞间信息交流的重要媒介,与多种疾病的发生发展密切相关。在复杂体系中开展外泌体分离富集研究,对于疾病诊断、预后检测和分子机制研究具有重要意义。但外泌体广泛存在于复杂生命体系中,尺寸小且分布范围宽,为其分离富集带来了困难与挑战。目前已经发展了基于物理性质的超速离心、体积排阻色谱、超滤和聚合物沉淀等方法。以亲和配体为识别单元,基于分子识别的亲和分离策略能够实现外泌体高选择性分离富集。在众多分子识别工具中,多肽凭借着可设计性强、构象自由灵活等优势,被应用于外泌体亲和分离之中。本文首先对目前外泌体分离富集方法进行了总结,之后重点介绍了以外泌体蛋白质和生物膜特性为靶标的亲和多肽设计与筛选原理及其在外泌体亲和分离富集中的应用。

## Abstract (en)

Exosomes are bilayered vesicles derived from living cells and bacteria that are loaded with abundant biomolecules, such as proteins and nucleic acids. As an important medium of remote cell communication, exosomes are closely related to the occurrence and development of a number of diseases, including those involving tumors and inflammation. The isolation and enrichment of exosomes in complex biosystems is greatly significant for the diagnosis, prognosis, and detection of diseases, as well as in molecular-mechanism research. However, exosomes are usually nanoscale size distribution and widely existed in complex biological samples, including blood, tissue fluids, and urine, which bring difficulties and challenges to the isolation and enrichment of exosomes. To address this issue, several methods based on the physical properties of exosomes have been developed. For example, exosomes can be obtained by ultracentrifugation at high centrifugal force based on density differences; they can also be isolated and enriched by size-exclusion chromatography and ultrafiltration based on size heterogeneity. Exosomes can also be separated in high yields but with low purities using commercial polymer-coprecipitation-based isolation kits. While the abovementioned methods can be used to isolate and enrich exosomes in a highly efficient manner, accurately distinguishing interfering particles, including protein aggregates and microvesicles, in biosystems is still difficult, resulting in the poor purity of exosome isolation and enrichment. Affinity ligands are widely used during the affinity isolation and enrichment of exosomes. Antibodies exhibit high selectivity and affinity; consequently exosomes can be captured highly selectively by exploiting specific antigen/antibody interactions. However, antibodies also have some limitations, including complex preparation procedures, high costs, and poor stability. Chemical affinity ligands, such as aptamers, peptides, and small molecules, are also widely used to isolate and enrich exosomes. As a kind of molecular recognition tool, peptides contain a variety of amino acids and exhibit many advantages, including good biocompatibility, low immunogenicity, and design flexibility. Solid-phase synthesis strategies have rapidly developed, thereby providing a basis for automated and large-scale peptide synthesis. Affinity peptides have been widely used to recognize and analyze target biomolecules in complex physiological environments in a highly selective manner. A series of protein-targeting peptides has been reported based on the biomolecular characteristics of exosomes. These affinity peptides can be specifically anchored onto highly enriched transmembrane proteins on exosome surfaces, thereby enabling the efficient and highly selective isolation and enrichment of exosomes in complex systems. Additionally, exosomes contain stable bilayer membranes consisting of abundant and diverse phospholipid molecules. The development of phospholipid-molecule-targeting peptides is expected to effectively eliminate interference from protein aggregates and other particles. In addition to differences in the compositions of phospholipids in biofilms, exosomes are smaller and more curved than apoptotic bodies and microvesicles. A series of affinity peptides capable of inducing and sensing high membrane curvatures are widely used to isolate and enrich exosomes. The tumor microenvironment can produce and release tumor-derived exosomes that are buried in a large number of normal cell-derived exosomes. Accordingly, pH-responsive peptides have been designed and modified based on the acidic environments of tumor-derived exosomes, which were accurately and tightly anchored via peptide insertion and folding. Focusing on the current status of exosome research, this paper introduces and summarizes current and widely used methods for isolating and enriching exosomes. Various exosome-targeting peptide-design and screening principles are introduced based on the characteristics and advantages of peptides. The applications of these peptides to the isolation and enrichment of exosomes are also summarized, thereby providing strong guidance for the efficient and highly selective isolation and enrichment of exosomes in complex life-related systems.

外泌体是由活细胞分泌的纳米级囊泡,尺寸通常为40~200 nm,携带有丰富的蛋白质、核酸和脂质等生物分子,是细胞间进行物质、信息交流的重要途径,调节着远程细胞通讯、免疫反应、细胞生长和分化等重要生命过程^[1,2]^。研究表明,外泌体的数量、表型以及分子特征反映了亲本细胞的生理病理状态,与癌症、炎症和神经退行性疾病等多种疾病的发生和发展密切相关^[3]^。因此,对外泌体的深入研究,将有助于拓展外泌体在生命医学和疾病诊断等领域的应用,为重大疾病的早期诊断和精准治疗提供有力指导。

高效、高选择性分离富集外泌体是进行下游分析研究的重要前提。然而,外泌体广泛存在于血液、尿液和脑脊液等复杂生物流体中,存在着严重的基质干扰^[4]^。同时外泌体也具有高度复杂的异质性,尺寸小且分布范围宽,结构组成丰富多样,为外泌体的高效、高选择性分离富集带来了巨大的挑战^[5]^。目前,复杂生命体系中外泌体分离富集方法包括超速离心^[6]^、体积排阻色谱^[7]^、超滤^[8]^和聚合物沉淀^[9]^等方法。但是这些方法主要基于外泌体的密度、尺寸或溶解度等物理性质,很难有效区分生命体系中的脂蛋白聚集体等干扰颗粒^[10]^。为了进一步提高外泌体分离富集的选择性,研究者们以外泌体表面的分子标志物为靶点,以亲和识别配体为靶向单元,发展了基于分子识别的亲和分离策略^[11]^,并将抗体、核酸适配体、多肽和小分子化合物等分子识别元件与种类广泛、设计灵活的分离介质相结合,构建了外泌体亲和分离新材料,以实现特定亚型外泌体的高选择性分离富集^[12]^。

靶向多肽作为分子识别元件,具有稳定性高、性质多样、作用力可控等优势^[13]^。固相多肽合成策略的迅速发展也为多肽自动化批量合成和位点特异性修饰提供了有效手段^[14]^。近年来,研究者们以分子标志物为靶点,设计、构建候选肽文库,通过亲和筛选获得了众多性质迥异的靶向多肽,实现了多肽识别导向的高选择性分离分析^[15]^。

围绕外泌体分离富集的现状与难题,本论文首先总结了在外泌体领域中已广泛应用的分离富集方法,主要包括基于外泌体物理性质和基于分子识别的分离方法(表1);并围绕靶向多肽的特点与优势,重点介绍了各种类型外泌体靶向多肽的设计与筛选原理及其在外泌体分离富集研究中的应用,以期为复杂生命体系外泌体的高效、高选择性分离富集提供信息。

**表1:** 外泌体分离富集方法的比较

Method	Mechanism	Advantages	Disadvantages
Ultracentrifugation	density	standard protocol, large sample volume	low yield and purity, ultracentrifuge
Size-exclusion chromatography	size	high reproducibility, fast and easy preparation	relatively small volume, low specificity
Ultrafiltration	size	fast and easy preparation, serviced in series	possibility of clogging, low specificity
Polymer precipitation	solubility	simple equipment and operation, high yield	low purity, long processing time
Affinity capture	molecular recognition	high specificity, small volume for rare sample	high cost, extra steps for exosome elution, optimization for exosome markers

## 1 外泌体分离富集方法

### 1.1 基于物理性质的外泌体分离富集方法

超速离心法是目前应用最为广泛的外泌体分离富集方法之一,在逐渐升高的离心力条件下,依次去除复杂生物流体中的悬浮细胞、细胞碎片和微囊泡,最终在超速离心力(100000 g)下,从样本中沉降并富集外泌体^[16,17]^。为了提高分离纯度,研究者们进一步发展了密度梯度离心法,在不同浓度的蔗糖或碘克沙醇溶液中进行离心,外泌体将保留在密度为1.13~1.21 g/cm^3^的平衡区域,从而与干扰物质有效分离,提高外泌体分离纯度^[18]^。然而,超速离心法依赖昂贵且费时费力的超速离心机,并且重复的超速离心过程也会对囊泡造成不可逆的损伤,降低分离产率与纯度^[19]^。

外泌体的尺寸通常大于多肽、蛋白质和核酸等生物分子的尺寸,体积排阻色谱和超滤法能够依据外泌体尺寸差异实现分离^[20]^。在凝胶柱中,尺寸超过凝胶孔径的外泌体无法进入凝胶内部,随着流动相快速流出;而生物分子可以进入凝胶空隙,保留时间延长,从而实现与外泌体的分离^[21]^。对外泌体膜进行荧光标记后,进一步将体积排阻色谱与荧光检测器联用,能够实现对外泌体的定量检测^[22]^。此外,体积排阻色谱具有良好的重现性,能够有效去除样本中杂质蛋白的干扰,并且多维体积排阻色谱的发展也为外泌体尺寸异质性分析提供了有力的工具^[23]^。

超滤法则是使用均一尺寸多孔膜截留生物样本中的外泌体,聚醚砜^[24]^、聚碳酸酯^[25]^、纤维素^[26]^和氧化铝^[27]^是常用的多孔膜材料。将孔径为200、100、80、50、30 nm的膜过滤器串联使用,可以实现对血浆、肺泡灌洗液和尿液等复杂样本中不同尺寸分布外泌体的分离富集^[25]^。但是滤膜孔径通常较狭窄,在分离生物样本时容易发生堵塞,使外泌体受压变形并破损。施加声波周期振荡^[27]^或采用切向流动^[24]^等手段减少层积垢和颗粒沉积,能够有效解决这一难题。

基于聚合物的共沉淀法已经发展为多款商品化外泌体分离试剂盒,聚乙二醇是应用最为广泛的沉淀试剂。亲水性聚乙二醇分子与外泌体周围的水分子相互作用,在囊泡四周形成疏水微环境,降低了外泌体的溶解度,通过低速离心处理即可实现外泌体的分离富集^[28]^。聚合物沉淀试剂盒所分离外泌体的产量高,避免了复杂的超速离心操作,维持了囊泡的完整性,但沉淀过程会使蛋白质、核酸和脂质等生物分子共沉淀,大大降低了外泌体分离富集的纯度^[29]^。因此,发展一种高纯度、高产率的外泌体分离试剂盒具有十分重要的意义。

### 1.2 基于分子识别的外泌体分离富集方法

外泌体广泛分布于复杂的生物系统中,其中存在着脂蛋白聚集体、微囊泡、凋亡小体等诸多干扰颗粒,仅依据外泌体密度、尺寸、溶解度等物理性质进行分离通常存在着特异性不足、纯度较低的局限性。因此,基于分子识别的亲和分离策略得到了越来越广泛的应用。

作为一种经典的识别工具,抗体具有高选择性、高亲和力的优势。对设计性和操作性灵活的磁球、微纳材料表面进行抗体修饰,基于抗原-抗体的特异性识别,能够实现复杂样本中外泌体的高选择性捕获^[30,31]^。抗体在发挥高选择性识别功能的同时,也存在着制备流程复杂、成本昂贵、稳定性较低等局限性。利用化学方法所构建的核酸适配体、靶向多肽和小分子化合物等识别配体,具有结构可设计、作用力可控、合成流程简便、稳定性高、靶标范围广等优势,在外泌体分离富集中得到了越来越多的应用^[32,33]^。Li等^[34]^将CD63适配体功能化微球与血清样本混合,样本中的外泌体被CD63适配体捕获到微珠表面,随后利用红外激光对体系进行局部加热,使携带有外泌体的微球发生定向热泳并富集,实现了乳腺癌患者血清中外泌体的分离富集。Li等^[35]^设计合成了磷脂酰胆碱功能化磁球,通过与外泌体膜表面磷脂分子产生静电配位相互作用,实现了4种侵袭程度不同的结直肠癌细胞培养液中外泌体的高产率、高纯度分离富集与蛋白质组学分析,为癌症转移相关新型标志物的鉴定提供了新的思路。

## 2 多肽识别导向的外泌体分离富集策略

由性质多样的氨基酸所构成的多肽亲和识别分子,不仅具有生物相容性好、免疫原性低等优势,而且可设计性强,易于合成与修饰,已被广泛应用于复杂生理环境中靶标生命分子的高选择性识别与分析检测^[36]^。针对外泌体的分子特征,目前已经发展了一系列以蛋白质为靶标的靶向多肽,此类多肽通常能够特异性锚定至外泌体表面高度富集的跨膜蛋白,实现复杂体系外泌体的高效、高选择性分离富集。此外,外泌体具有稳定的磷脂双分子层膜结构,基于生物膜的化学组成特征和生物物理性质,研究人员也发展了一系列磷脂靶向肽、曲率靶向肽、pH感应肽等新型多肽识别分子。围绕多肽识别靶标单元的不同,本节介绍了各类靶向多肽的设计与筛选原理及其在外泌体分离富集中的应用(表2)。

**表2:** 基于靶向多肽的外泌体分离富集方法

Peptide	Sequence	Targets	Samples	References
CP05	CRHSQMTVTSRL	CD63	serum	[37]
P238	RSHRLRLH	CD9	cell culture	[38⇓-40]
			mediums (CCMs)	
APQQ	RGLLVSQLQIQQ	CD81	unreported	[41]
Vn96	PSQGKGRLSLSRFSWGALTLGEFLKL	heat shock protein	CCMs, plasma,	[42⇓-44]
			cerebrospinal fluid	
PS-specific	CLIKKPF	phosphatidylserine	serum	[45]
peptide				
BK	RPPGFSPFR	membranes (curvature sensing)	CCMs	[46]
D-S v1	GGEQNPIYWARYADWLFTTPFGLLDLAHLVAADEGT	membranes (low pH-sensing)	serum	[47]

### 2.1 四次跨膜蛋白靶向多肽

作为一类外泌体经典标志物,四次跨膜蛋白(CD9、CD63和CD81)参与了囊泡形成、转运与货物分选过程,在外泌体表面高度富集^[48]^。四次跨膜蛋白在磷脂膜外侧存在着胞外大环和胞外小环两个结构域,适合于亲和配体的识别与结合,是外泌体分离富集的理想靶点^[49]^。Gao等^[37]^选择CD63蛋白的胞外大环作为靶点,通过噬菌体展示技术筛选获得了对外泌体具有高选择性、高亲和力的锚定肽CP05。将CP05固定在磁球表面,实现了人血清样本中外泌体的高效分离富集,每毫升血清中外泌体捕获量可达(108.98±7.82) μg。基于CP05对外泌体优异的锚定性能,研究者们进一步设计合成了一系列多肽-药物缀合物,通过简单、温和孵育即可在外泌体表面高效负载多种药物分子,成功应用于肌营养不良^[37]^、组织损伤^[50]^、肿瘤^[51]^、增生性视网膜病变^[52]^、外伤性视神经病变^[53]^等诸多疾病的靶向治疗。

研究表明,四次跨膜蛋白CD9与其伴侣蛋白EWI-2存在着特异性相互作用,形成紧密的四聚体复合物^[54]^。Suwatthanarak等^[38]^从生命体系中天然存在的CD9-EWI-2相互作用出发,设计并合成了基于微孔阵列的亲和筛选平台。将完整的EWI-2蛋白分解为八肽片段,设计并化学合成了容量为270的候选肽文库,通过测定其与荧光标记外泌体孵育后微孔中的荧光强度,筛选获得了一条对MDA-MB-231乳腺癌外泌体具有最高亲和力的靶向多肽P238,平衡解离常数(*K*_D_)为4.66×10^-7^mol/L。进一步对P238进行了单位点和多位点丙氨酸残基置换,探索P238与CD9的结合机理^[39,40]^。当精氨酸、组氨酸和丝氨酸替换为丙氨酸后,P238对CD9蛋白的结合能力显著降低,表明静电和极性相互作用在多肽-蛋白质识别中发挥了重要作用。研究者们进一步将P238与氧化锌结合序列偶联,锚定于氧化锌纳米线阵列表面,实现了材料表面靶向多肽的高效修饰,修饰量为(1.15±0.06) nmol/mm^2^。基于多肽-跨膜蛋白优异的识别性能,多肽功能化氧化锌纳米线阵列实现了细胞培养液中外泌体的高效捕获,捕获效率是超速离心法的3.8倍。

针对四次跨膜蛋白CD81缺少天然结合配体的现状,本课题组从正义肽-反义肽相互作用原理出发,开展了CD81蛋白靶向多肽的设计、筛选与应用研究(图1)^[41]^。选择CD81蛋白胞外小环中构象自由延展的十一肽片段作为正义肽,基于亮氨酸遗传密码子简并性原理,对直读反义肽进行了反义氨基酸残基替换,设计并合成了候选反义肽。在正义肽N段修饰具有聚集诱导发光效应的四苯乙烯分子后,以荧光发射强度指示体系中存在的正义肽-反义肽相互作用强度,筛选获得了对CD81蛋白具有最强结合性能的新型反义肽APQQ(*K*_D_为(318±33.6) nmol/L)。利用新型反义肽作为分子探针,发现并解析了CD81蛋白在细胞连接区域的靶向聚集行为,实现了CD81蛋白表达响应的肿瘤细胞迁移抑制,成功应用于高侵袭性三阴性乳腺癌肺转移的高效靶向抑制。基于APQQ具有位点特异性识别并稳定结合CD81蛋白胞外小环的特点,该新型反义肽有望应用于复杂生命体系中以CD81蛋白为靶标的外泌体亲和分离富集。

**图1:**
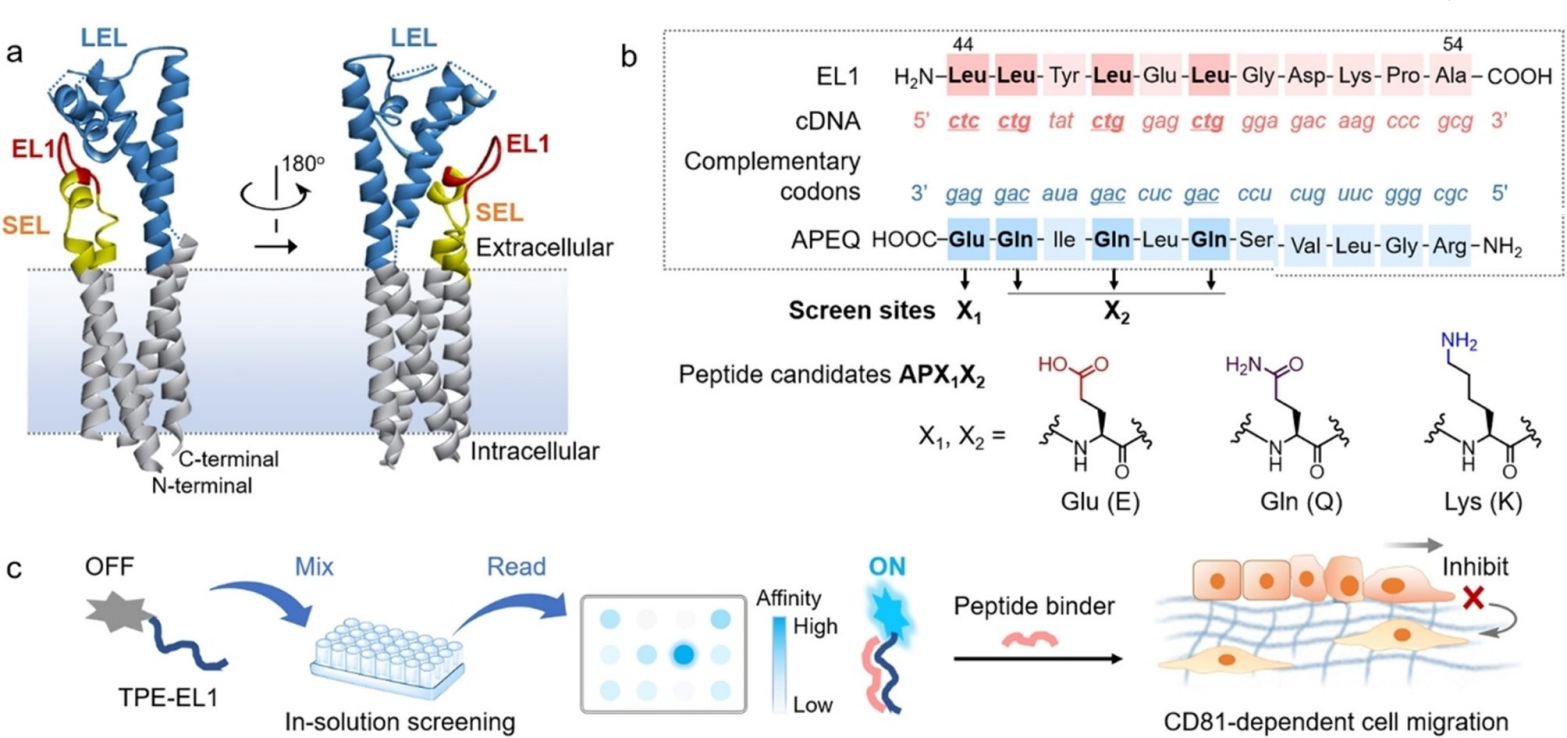
基于正义肽-反义肽相互作用原理的CD81靶向多肽的设计与亲和筛选^[41]^

### 2.2 热休克蛋白靶向多肽

热休克蛋白(HSP)是热应激反应过程中合成、分泌的一类重要蛋白质,与蛋白质折叠、跨膜转运和降解等过程密切相关^[55]^。作为一种膜蛋白,HSP广泛分布于胞内腔室和外泌体等膜结构区域,在外泌体中高度表达。研究表明,HSP的C端存在着具有疏水结合口袋的底物结合域,与具有疏水侧链的阳离子型多肽具有强烈的结合倾向^[56]^。Ghosh等^[42]^设计合成了一系列长度为20~30个氨基酸残基、富含疏水侧链的阳离子型候选肽,通过反向迁移等电聚焦技术考察了候选肽与HSP形成复合物的能力,筛选获得了一条与HSP具有最高结合性能的靶向多肽Vn96(图2)。

**图2:**
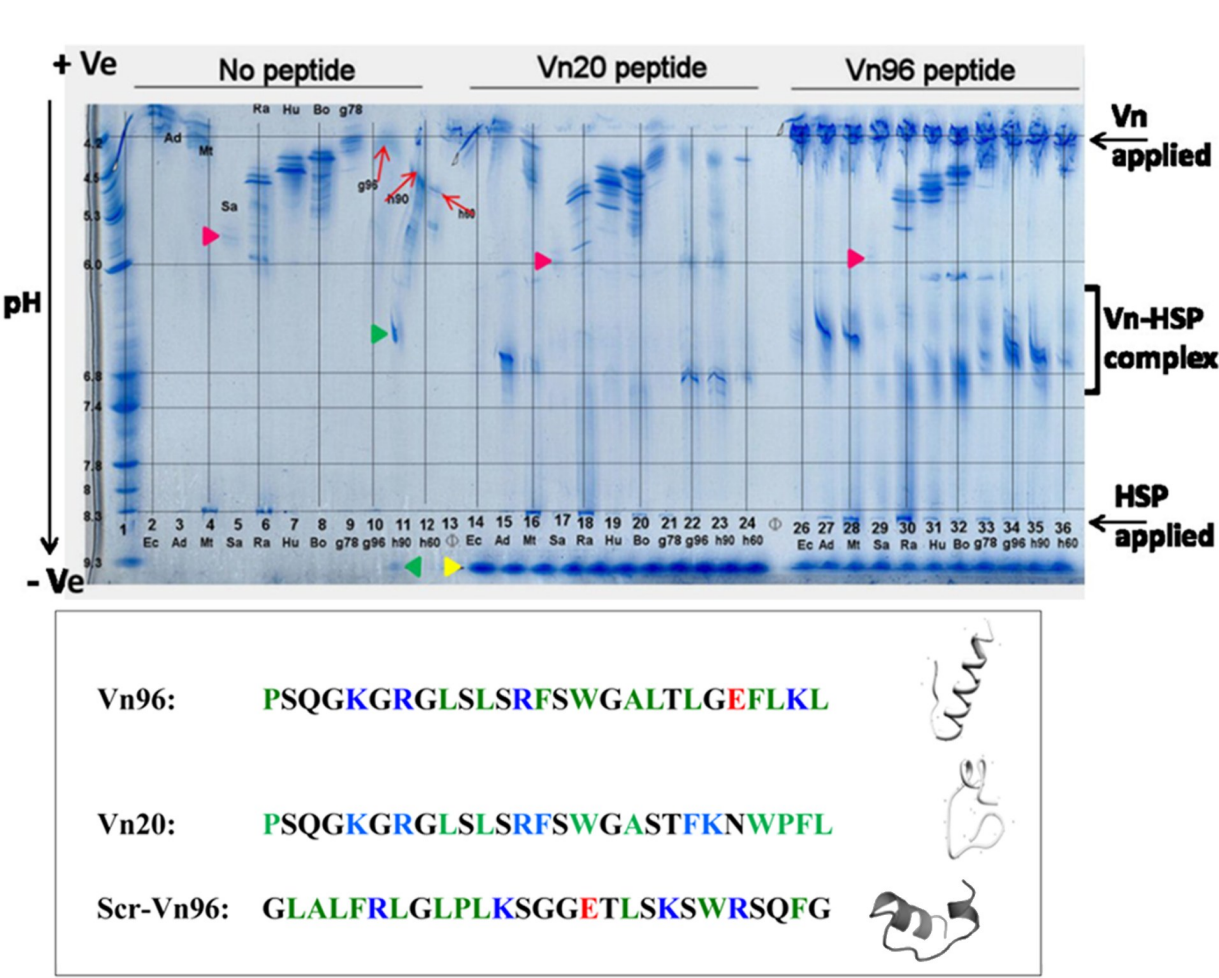
反向迁移等电聚焦用于重组HSP亲和多肽的筛选^[42]^

多肽Vn96以阳离子α螺旋形式高效锚定至外泌体膜表面的HSP,提高了外泌体表面的疏水性并促使其聚集,通过低速离心即可实现细胞培养液和生物体液中外泌体的高效分离富集。通过与经典的超速离心法进行比较,使用Vn96肽能够在1.5 h内实现外泌体的快速、高产率和高纯度分离富集,两种方法所鉴定的蛋白质种类和数目存在着85%的重叠^[43]^。基于Vn96与HSP优异的识别性能和引发外泌体聚集沉淀的特性,Vn96已被应用于阿尔茨海默病患者脑脊液中外泌体的亲和分离富集与下游蛋白质组学分析^[44]^,也被发展成为了一款商业化外泌体分离试剂盒,其分离纯度显著高于目前的聚乙二醇沉淀试剂盒。

### 2.3 磷脂靶向多肽

外泌体是具有双层膜结构的纳米级囊泡,在外泌体膜上存在着丰富多样的磷脂分子,例如磷脂酰丝氨酸、磷脂酰乙醇胺、鞘磷脂等。在外泌体形成过程中磷脂层发生重新排布与分配,磷脂酰丝氨酸在外翻酶的作用下发生外化,使得其在外泌体膜外侧高度富集^[5]^,是外泌体分离富集的理想靶点,基于磷脂酰丝氨酸分子印迹技术的亲和分离方法也不断发展^[57,58]^。除此之外,发展以磷脂分子为识别导向的磷脂靶向肽,能够有效排除蛋白质聚集体等颗粒的干扰,实现外泌体的高效、高选择性分离富集^[59]^。Yang等^[45]^通过噬菌体展示技术筛选获得了磷脂酰丝氨酸靶向多肽(CLIKKPF),通过点击反应将其固定于二氧化硅微球表面,构建了多肽功能化外泌体亲和分离微球(图3)。通过固定化多肽配体与磷脂酰丝氨酸的特异性相互作用,在2 h内以高产率、高纯度的特点实现了健康志愿者、肝癌患者和肝内胆管癌患者血清外泌体的分离富集。

**图3:**
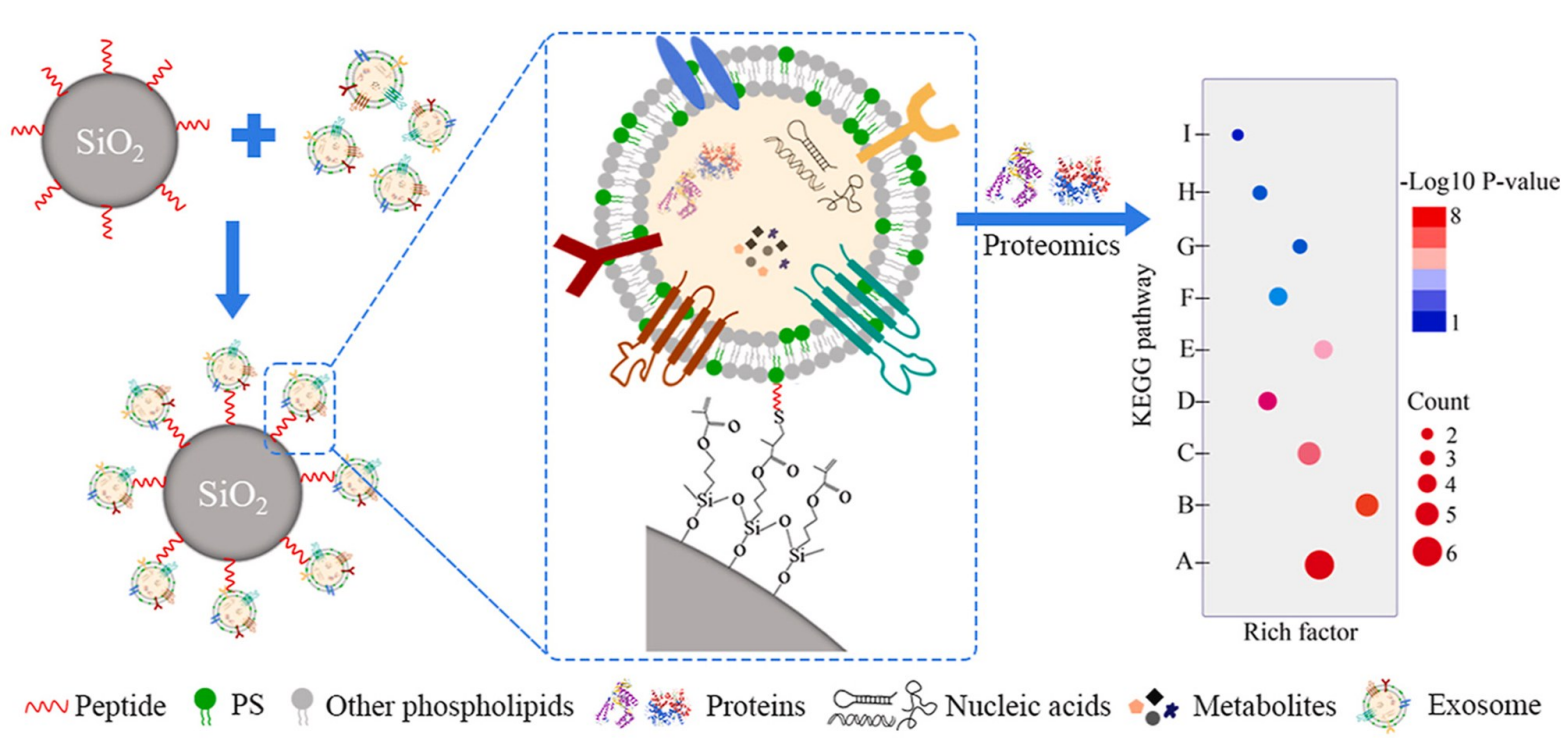
磷脂酰丝氨酸亲和多肽功能化SiO_2_微球用于外泌体分离和蛋白质组学分析^[45]^

### 2.4 曲率靶向多肽

与凋亡小体、微囊泡相比,除了生物膜上磷脂分子组成存在差异之外,外泌体还具有更小的尺寸和更大的弯曲程度。因此,高曲率也是纳米级外泌体区别于大尺寸囊泡的独有特性。一系列能够诱导和感知膜曲率的靶向多肽也广泛应用于外泌体的分离富集与识别检测^[60]^。例如,跨膜突触蛋白Syt-1是一种调节膜转运和融合过程的蛋白,其环状L3结构域(363~372位氨基酸)能够嵌入磷脂双层膜之中,具有强烈的膜曲率识别性能^[61]^。Saludes等^[61]^通过固相合成策略制备了L3线性片段,通过点击反应将多肽N端与C端相连,模拟自然界中L3结构域的环状结构,设计合成了具有膜曲率感知性能的环肽探针,对尺寸小于100 nm的囊泡具有选择性识别能力。

缓激肽(BK)是一种体内存在的九肽激素,富含精氨酸等正电荷氨基酸,与负电性磷脂膜具有高度的结合倾向^[62]^。当缓激肽与脂质作用时,其二级螺旋结构有助于多肽嵌入磷脂层中,加强了与磷脂膜的相互作用^[63]^。Gori等^[46]^以缓激肽为识别单元,设计合成了键合有单价、串联二价以及并联二价型缓激肽的外泌体分离芯片,与商业化Exoview检测仪器联用,无需预处理即可完成细胞培养液和血清中外泌体的分离富集,实现了外泌体表面四次跨膜蛋白的成像分析(图4)。值得注意的是,曲率靶向肽凭借其α螺旋嵌入磷脂膜之中,实现对外泌体的锚定与识别,但是当多肽浓度超过特定临界浓度时,曲率靶向肽通常会诱导囊泡脂质膜上产生孔道,从而引发囊泡解离^[64]^。

**图4:**
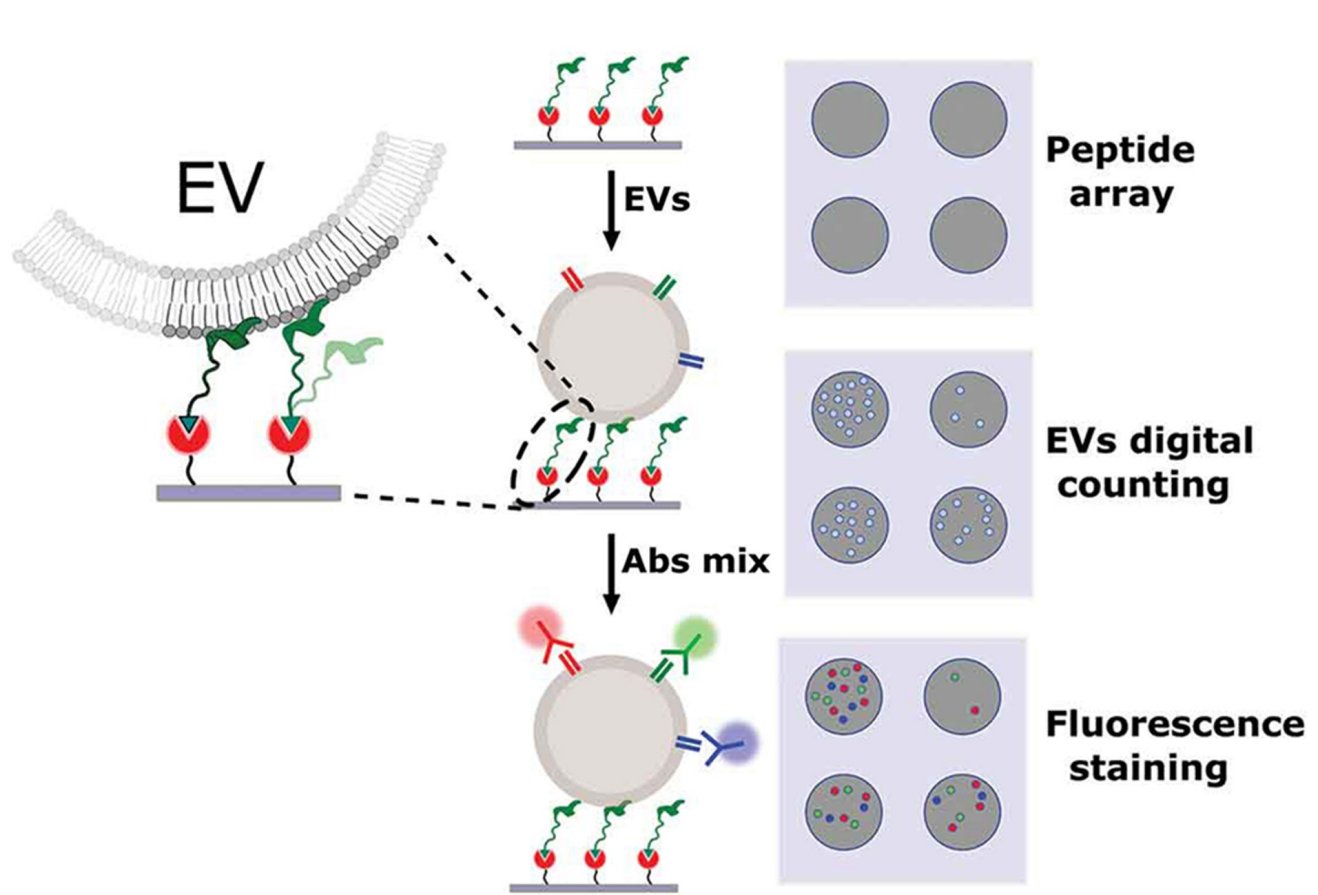
基于曲率靶向肽的外泌体分离与荧光检测示意图^[46]^

### 2.5 pH响应多肽

肿瘤微环境是由肿瘤细胞、上皮细胞和细胞外基质等多种成分组成的微系统,具有缺氧、微酸性等特点^[65]^。肿瘤微环境中存在着大量肿瘤来源外泌体,进入循环系统后被掩盖于众多非肿瘤来源外泌体之中,为肿瘤外泌体的高选择性分离富集带来了挑战。针对肿瘤微环境的酸性特征,Liu等^[47]^以细菌视紫红质的第三跨膜螺旋区为先导肽,开展了酸性pH响应多肽的设计、筛选与外泌体分离富集研究(图5)。处于酸性微环境的先导肽会产生折叠并嵌入磷脂膜中,实现对肿瘤微环境来源外泌体的选择性标记。为了避免正常生理pH环境中先导肽的解折叠,对先导肽进行多位点氨基酸突变,以改变第14和25位天冬氨酸所处微环境。通过分子动力学计算与筛选,新型低pH嵌入肽(D-S v1)能够通过形成稳定的钩状空间结构,不可逆地锚定至肿瘤外泌体膜中。进一步对多肽D-S v1的末端偶联生物素分子,当多肽嵌入磷脂双分子层后,利用链霉亲和素功能化磁球实现了肺癌小鼠血清中肿瘤来源外泌体的选择性捕获。

**图5:**
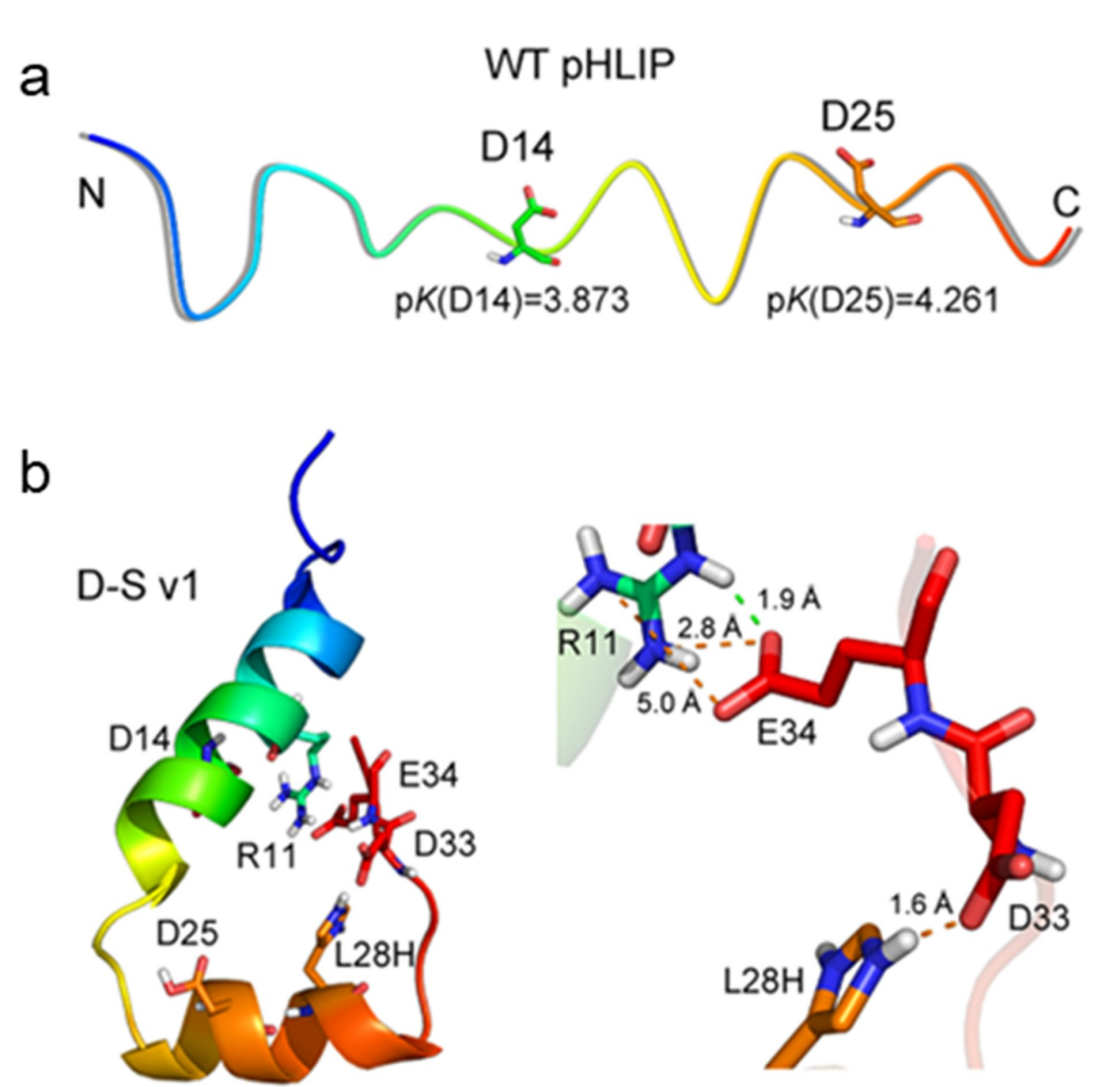
低pH嵌入肽(pHLIP)的结构和折叠行为^[47]^

## 3 结论

近年来,随着对外泌体的深入、广泛研究,研究者们发展了基于不同原理的分离富集方法,实现了生物体液中外泌体的分离富集,在分离产率和纯度、操作效率等方面取得了很大进展。针对外泌体的尺寸、密度和溶解度等物理性质,建立了超速离心、体积排阻色谱、超滤和聚合物沉淀等多种分离方法,但存在着特异性不足的局限性。基于分子识别的亲和分离策略能够有效排除蛋白聚集体等颗粒的干扰,提高外泌体分离富集的纯度。然而,每种方法都有其固有的优势与局限性,难以依靠单一分离方法实现外泌体的高效、高选择性分离富集。因此,针对特定的研究目标,合理选择并联合现有的分离方法,或发展外泌体分离富集新方法对于外泌体分离富集具有重要意义。例如,微流控技术具有分离速度快、所需样品量少等独特优势,对通道结构进行合理设计及表面亲和配体功能化,能够显著提高微量流体中外泌体亲和分离的效率与纯度。切向流超滤系统及场流分离系统也广泛应用于不同尺寸囊泡的分离富集,为外泌体尺寸异质性分析提供了有力工具。利用红外激光对体系进行局部加热,使囊泡定向热泳并富集,也逐渐成为一种新兴的囊泡分离分析技术。除此之外,靶向多肽凭借着结构可设计、作用力可控、稳定性高、易于化学合成与修饰等优势,在外泌体高选择性分离分析中得到了广泛的应用。以外泌体表面高度表达的膜蛋白为靶标,发展了一系列蛋白质靶向多肽,通过与种类广泛、设计灵活的分离材料相结合,实现了特定亚型外泌体的高选择性分离。基于外泌体生物膜的化学组成与高曲率特性,磷脂靶向肽和曲率感应肽也逐渐应用于外泌体分离富集。为了选择性识别肿瘤微环境所分泌的外泌体,根据其所处酸性pH特点,设计并改造了pH响应多肽,实现了肿瘤外泌体的精准锚定与分离富集。虽然目前已有不同类型的外泌体靶向多肽被报道并应用,但是多肽配体与靶标分子的亲和力与选择性仍需优化与提升,且靶标分子在外泌体表面的表达量存在显著差异,这也极大地限制了多肽在外泌体分离分析中的应用。因此,从自然界中存在的多价协同识别原理出发,发展新型协同多肽识别分子,构建协同多肽功能化分离新材料,探索分离过程中亲和界面与多靶标的协同作用规律,将有望为外泌体分离分析提供新工具和新方法。
